# The Importance of Thioredoxin-1 in Health and Disease

**DOI:** 10.3390/antiox12051078

**Published:** 2023-05-11

**Authors:** Tina Oberacker, Leonie Kraft, Moritz Schanz, Jörg Latus, Severin Schricker

**Affiliations:** 1 Dr. Margarete Fischer-Bosch Institute for Clinical Pharmacology, 70376 Stuttgart, Germany; 2 Department of Internal Medicine and Nephrology, Robert-Bosch-Hospital Stuttgart, 70376 Stuttgart, Germany; leonie.kraft@rbk.de (L.K.); moritz.schanz@rbk.de (M.S.); joerg.latus@rbk.de (J.L.); severin.schricker@rbk.de (S.S.)

**Keywords:** thioredoxin, redox regulation, oxidative stress, antioxidants, trx inducers, trx inhibitors

## Abstract

Thioredoxin-1 (Trx-1) is a multifunctional protein ubiquitously found in the human body. Trx-1 plays an important role in various cellular functions such as maintenance of redox homeostasis, proliferation, and DNA synthesis, but also modulation of transcription factors and control of cell death. Thus, Trx-1 is one of the most important proteins for proper cell and organ function. Therefore, modulation of Trx gene expression or modulation of Trx activity by various mechanisms, including post-translational modifications or protein–protein interactions, could cause a transition from the physiological state of cells and organs to various pathologies such as cancer, and neurodegenerative and cardiovascular diseases. In this review, we not only discuss the current knowledge of Trx in health and disease, but also highlight its potential function as a biomarker.

## 1. Introduction

Thioredoxin-1 (Trx-1) is a 12 kDa multifunctional protein localized mainly in the cytosol and in some cases in the nucleus [1]. It was first mentioned in the 1960s as a hydrogen donor for enzymes in yeast that reduce methionine sulfoxide [2] and sulfate [3] through nicotinamide adenine dinucleotide phosphate (NADPH). In 1964, Trx-1 was isolated from *E. coli* as an electron donor for ribonucleotide reductase [4]. Human Trx was independently cloned as adult T-cell leukemia derived factor (ADF) from the supernatant of a T-cell line infected with HTLV-I and initially designated as ADF [5].

Trx-1 is a protein that contains two redox-active cysteine residues (Cys32 and Cys35) in its active site (Cys-Gyl-Pro-Cys). These cysteines are responsible for its reducing activity, ensuring that the thiols of the substrates are maintained in a reduced state while being oxidized themselves [6]. In general, Trx-1 plays a critical role in a variety of cellular functions, such as proliferation, maintenance of redox homeostasis, DNA synthesis, regulation of gene expression, and control of cell death by apoptosis [6,7,8,9]. In addition, Trx-1 dysfunction has been shown to be associated with a variety of diseases including cancer, as well as neurodegenerative and cardiovascular diseases [10,11,12]. Moreover, Trxs are essential for mammalian development, as a deficiency of Trx-1 or Trx-2 is embryonically lethal in mice [13,14].

### 1.1. The Thioredoxin Family Proteins

**Figure 1:**
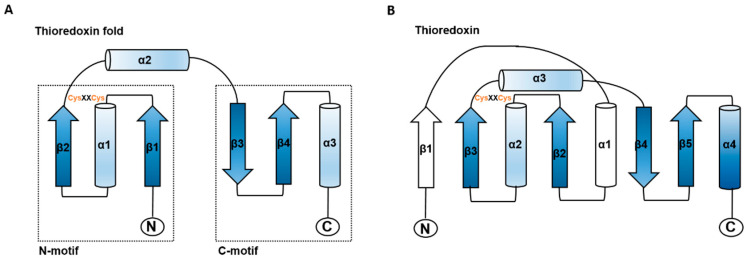
Schematic structure of the thioredoxin fold and *E. coli* thioredoxin. (**A**) The structural elements of the thioredoxin fold are shown in blue. (**B**) The structure of *E. coli* thioredoxin is shown with the typical thioredoxin fold in blue and additional features in white. The location of the CysXXCys motif and N-and C-termini are indicated. Arrows represent ß-strands, and α-helices are shown as cylinders (adapted from Martin, 1995 [17]).

The thioredoxin superfamily is an ancient protein family [15]. All members of this superfamily share an active site with -Cys-X-X-Cys- (X can be another amino acid) and four-stranded β-folds and flanking α-helices in the secondary structure (Figure 1) [16,17]. This structural motif has been identified in Trxs, glutaredoxins (Grxs), glutathione S-transferase, disulfide bond formation protein A (DsbA), glutathionine peroxidases [17], peroxiredoxin (Prx), arsenate reductase (ArsC), quiescin sulfhydryl oxidases (QSOX), and vitamin K epoxide reductase (VKOR) [18]. Although Trx family members exhibit a diversity of functions and structures, most members are involved in redox regulation.

### 1.2. The Thioredoxin System

The Trx system and the glutathione system are among the most important thiol reduction systems [19]. In general, the thioredoxin system is a family of cysteine-dependent antioxidant proteins that includes NADPH, thioredoxin reductase (TrxR), and Trx-1 itself [19,20]. These three components form a large disulfide reductase system that provides electrons to various proteins. 

The interaction of these components contributes to the maintenance of the cellular redox balance [21] and regulates a variety of cellular functions, including transcription, DNA synthesis, and stimulation of cell growth [7]. 

In their active site, thioredoxins possess the so-called CGPC motif, which is responsible for catalyzing the protein disulfide/dithiol exchange. The CGPC motif is also used by many other enzymes involved in thiol-dependent anti-oxidative defense, such as glutaredoxin, peroxiredoxin, and glutathione peroxidase [22,23,24].

In mammals, there are at least three types of Trx: cytosolic thioredoxin (Trx-1), mitochondrial thioredoxin (Trx-2), and sperm Trx (Sp-trx or p32^TrxL^). An extracellular truncated form of thioredoxin containing the 80 N-terminal residues (Trx80) is known to be secreted from monocytes and CD4^+^ T cells [25]. 

## 2. Functions of Trx-1

**Figure 2:**
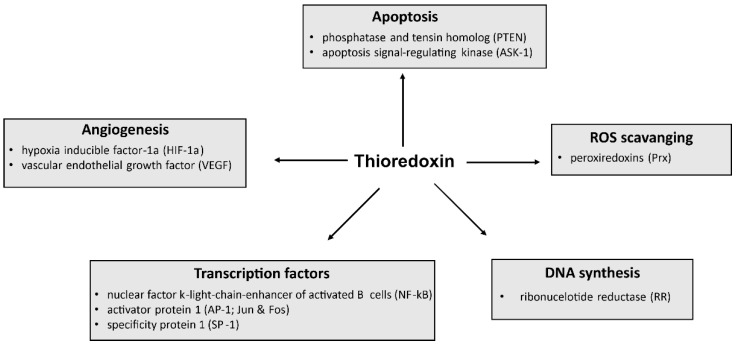
Overview of cellular functions and proteins affected by Trx.

Generally, Trx-1 plays an important role in various cellular functions, including maintenance of redox homeostasis, proliferation, and DNA synthesis, but also modulation of transcription factors and control of cell death (Figure 2) [6,7,8,9]. Moreover, Trx-1 plays a key role in several diseases, and high Trx-1 levels are associated with poor prognosis in certain diseases. Therefore, targeting Trx-1 also has clinical implications for disease treatment and prevention. 

### 2.1. Antioxidant Defense

An excess of reactive oxygen species (ROS) causes severe damage to lipids, proteins, and DNA [26]. To limit the effects of ROS, cells have evolved complex processes [27]. For example, so-called antioxidants play a central role in the cellular defense against ROS and can act either directly or indirectly.

**Figure 3:**
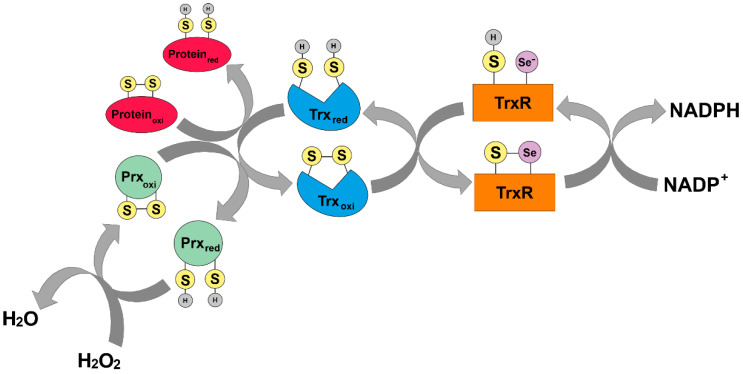
Mode of action of the thioredoxin system. Trx binds to oxidized proteins by reducing one thiol group through the formation of an intermolecular disulfide bridge. Subsequently, this mixed disulfide bridge is cleaved, also attacking the second thiol group in the protein. The second thiol group is reduced and both thiols within Trx are released from a disulfide bridge, rendering Trx inactive. TrxR recycles Trx in an NADPH-dependent manner (adapted from Karlenius and Tonissen, 2010 [29]).

In addition to glutathione, Trx is an endogenous direct-acting antioxidant [20]. The Trx system (see Section 1.2) can directly scavenge ROS and reduce disulfide bridges in oxidized proteins using its two redox cysteine residues (Cys32 and Cys35). In principle, Trx binds to oxidized proteins by reducing one of its thiol groups. In parallel, an intermolecular disulfide bridge is formed. Subsequently, this mixed disulfide bridge is cleaved and the second thiol group in the protein is also attacked. The second thiol group is reduced and both thiols within Trx are separated from a disulfide bridge, rendering Trx inactive. Trx is recycled in an NADPH-dependent manner by TrxR (Figure 3) [28].

In addition, cells can also utilize Prx family members as cellular antioxidants capable of scavenging hydrogen peroxide by using their peroxidic cysteine residues as electron donors and forming a disulfide bridge with their other reactive cysteine [30]. The intermolecular disulfide thus formed is then reduced by Trx-1, which in turn is reduced by TrxR.

#### 2.1.1. Regulation of Trx Activity

Trx activity can be modulated at various levels, including gene expression, post-translational modifications, or protein–protein interactions [31]. Through these mechanisms, the Trx system can respond to changes in the cellular environment. 

##### Gene Expression Level

The transcription factors (TF) nuclear factor erythroid 2-related factor 2 (Nrf2), TATA-binding protein (TBP), and cAMP response element-binding protein (CREB) can regulate the Trx system at the level of gene expression [32,33].

In general, various stressors, such as oxidative stress (OS) or inflammation, activate these transcription factors. After activation, the TFs bind to the so-called antioxidant response element (ARE) in the promotor region of Trx [34]. Interestingly, Trx in its reduced form promotes Nrf2 binding to ARE by reducing cysteine residues in the DNA-binding loop of small Maf proteins (sMaf), which in turn leads to activated Nrf2 transcription [34].

##### Protein–Protein Interaction 

The biological activity of the thioredoxin system depends on endogenous blocking inhibitors binding at least one of the components of the system. The thioredoxin-interacting protein (Txnip) is an example of such a binding partner. Txnip belongs to the α-arrestin family and was initially called Vitamin-D_3_-Upregulated-Protein 1 [35] because it is upregulated in human leukemia cells (HL60) treated with 1,25-(OH)_2_D_3_ [36].

Studies using the yeast two-hybrid system showed that Txnip binds to the catalytic center of Trx, thereby inhibiting its activity and expression [35,37]. Thus, Txnip, and Trx play an important role in maintaining redox homeostasis [37,38,39,40]. 

Substitution of cysteine by serine at the Cys247 position of Txnip failed to bind to Trx, indicating that this residue is necessary and sufficient for a mixed disulfide bridge with Trx, leading to the abolition of its inhibitory effect on Trx [39,41,42]. Therefore, the cysteine Cys247 of Txnip may be a potential therapeutic target in the future [43,44].

##### Post-Translational Modifications

In addition to its redox-active cysteine residues (Cys32 and Cys35), Trx has three non-active cysteine residues (Cys62, Cys69, and Cys73) that can be post-translationally modified, thereby altering its activity. Typical post-translational modifications of Trx include phosphorylation, S-nitrosylation, and glutathionylation [31]. 

Phosphorylation of Trx at threonine 100 (T100) affects its anti-apoptotic activity, as shown in HepG2 cells by lower stress-induced survival and a higher apoptosis rate induced by cis-platinum [45]. Therefore, abrogating the phosphorylation of Trx at T100 may be of interest for cancer therapy [45]. Another way to modify Trx activity is to glutathionylate Trx at Cyst73, preventing its dimer formation and abolishing its enzymatic activity [46].

Several reports have demonstrated that S-nitrosylation of Trx occurs at Cys62, Cys69, or Cys73 [47,48]. Haendeler and colleagues showed that S-nitrosylation of Trx at Cys69 is essential for its anti-apoptotic function, as it is the prerequisite for trans-S-nitrosylation of target proteins such as caspase-3 [48]. Moreover, nitrosylation at Cys73 can only occur after the formation of a disulfide bound between Cys32 and Cys35 [49].

### 2.2. Regulation of Gene Expression

In addition to its antioxidant function, Trx-1 can also activate oxidatively inactive transcription factors involved in redox regulation [50]. 

#### 2.2.1. Activator Protein 1 (AP-1)

Normally, Trx-1 is located in the cytosol [51], but in response to oxidative stress, Trx-1 is translocated to the nucleus. Trx-1 does not interact directly with AP-1 (a complex of *jun* and *fos* genes). However, Trx-1 activates AP-1 transcription by reducing the intermediate protein redox activating factor-1 (Ref-1), which then reduces a conserved cysteine residue in the DNA binding domain of Jun and Fos [52,53], thereby enhancing its DNA binding activity [54]. 

#### 2.2.2. Nuclear Factor Kappa-Light-Chain-Enhancer of Activated B-Cells (NF-κB)

The translocation of Trx-1 into the nucleus serves to enhance DNA binding of NF-κB [52,55]. NF-κB is critical for cytokine production and resides in the cytosol in an inactive protein complex bound to its inhibitor IκB. Various stimuli, such as excessive ROS production, activate the IKK pathway, leading to dissociation of NF-κB from its inhibitor IκB and translocation of NF-κB to the nucleus. 

The c-Jun N-terminal kinase (JNK) may play a role in IkB degradation and Trx-1 mediated activation of NF-kB [56]. Redox-active thioredoxin triggers the JNK signaling pathway, which is initiated at the MEKK1 level and leads to the activation of NF-κB through the degradation of IkB [56].

High levels of oxidative stress in the nucleus lead to oxidation of cysteine 62 (Cys62) in the p50 subunit of NF-κB, which impairs DNA binding. This oxidized cysteine in the p50 subunit can be reduced by Trx-1 by the formation of a multimolecular complex with NF-κB. The reduction of Cys62 allows NF-κB to bind to DNA, thereby, increasing gene expression of target genes [57,58,59].

### 2.3. Regulation of Cell Death

Apoptosis is an essential process during development, homeostasis, and the pathogenesis of various diseases. Under pathological conditions, apoptosis is inhibited by binding of apoptosis signal-regulating kinase 1 (ASK1) to reduced Trx-1 [60]. However, various stimuli such as tumor necrosis factor-α (TNF-α), endoplasmic reticulum stress, chemotherapeutic agents, and ROS can activate ASK1. Under pathological conditions, apoptosis is inhibited by binding of ASK1 to reduced Trx-1 by oxidizing Trx-1, leading to the release of ASK1, which is a prerequisite for the induction of apoptotic signals. Moreover, ubiquitination and degradation of ASK1 are induced by Trx-1 in a redox-dependent manner, which may lead to the inhibition of apoptosis [61]. In addition, apoptosis can be inhibited by Trx-1, which catalyzes the S-nitrosylation of procaspase-3 and caspase-3 [47,62,63].

### 2.4. Inflammation

Moreover, Trx plays an essential role in the activation, proliferation, and response of T cells, B cells, and macrophages [64], as immune cells can switch their redox system upon activation and activate the Trx signaling pathway. In addition, Trx-1 can exert anti-inflammatory effects due to inhibition of complement activation and macrophage migration inhibitory factors [65].

### 2.5. Trx-1 in Disease

**Figure 4:**
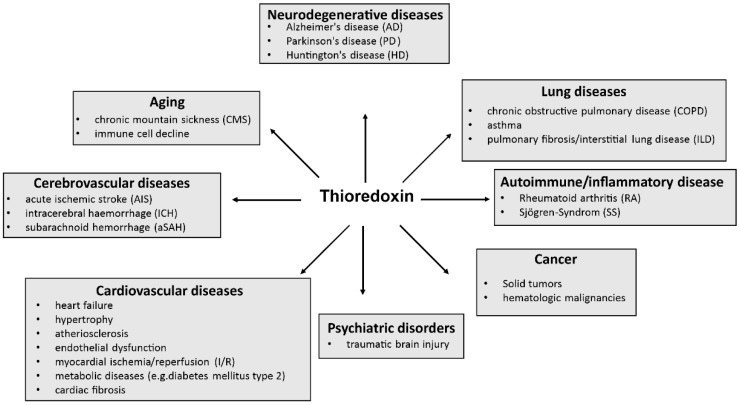
Overview of diseases influenced by Trx. The map shows that Trx-1 is involved in the pathogenesis of a variety of diseases.

It is known that Trx-1 is one of the most important proteins for proper cell and organ function. Therefore, it is not surprising that dysfunctional Trx-1 or altered expression of thioredoxin are involved in the development of a variety of diseases, including neurodegenerative diseases [10,66,67,68,69,70,71,72], lung diseases, autoimmune diseases, cancer, cardiovascular or cerebrovascular diseases, psychiatric disorders, and aging (see Figure 4). Notably, cancer analyses have shown that Trx-1 expression is increased in tumors [11,73], but Trx levels decrease when the tumors are surgically removed [74].

## 3. Trx as a Predictive Biological and Prognostic Marker in Cancer

Trx-1 is also essential for proper function of organs and tumors. Therefore, it is not surprising that more and more therapeutic approaches targeting Trx-1 are being developed. Since Trx is associated with many different diseases, especially cancer, it would be extremely interesting to know whether Trx can be used as a clinical and prognostic biomarker. Numerous studies have already been conducted on this topic, with promising results. 

### 3.1. Lung

Kakolyris et al. studied Trx-1 expression in normal lungs and primary non-small cell lung carcinoma (NSCLC). They found that Trx-1 expression in lung carcinomas was predominantly cytoplasmic, whereas expression was cytoplasmic in the respiratory bronchial epithelium, alveolar epithelium, and alveolar macrophages of normal lungs. Moreover, high Trx-1 expression was found to be associated with a high proliferation index [75]. Furthermore, immunohistochemical analysis of lung carcinomas revealed that proliferation measured by Ki67 staining was associated with high Trx expression. In addition, a significant association was found between low Trx expression and high cytoplasmic p53 reactivity, with higher Trx expression levels associated with a more aggressive tumor phenotype and potentially a worse outcome for the patients [75].

### 3.2. Gut 

Trx-1 is known to be overexpressed in patients with liver metastases from colorectal cancer. Trx-1 expression is an independent prognostic factor, with high expression associated with poor prognosis, as shown by multivariate analysis [76]. Serum levels of the redox-related genes Trx and manganese superoxide dismutase (MnSOD) are potential clinical biomarkers for predicting patient prognosis in hepatitis C virus-related hepatocellular carcinoma (HCC), as overall survival rate was lower in patients with high MnSOD levels and lower Trx levels [74,77]. In gallbladder carcinoma (GBC), nuclear expression of Trx-1 at the invasion front is associated with poor patient outcome [78]. In addition, one study investigated the potential of Trx-1 as a biomarker for predicting gastric cancer recurrence. It was demonstrated that patients with low Trx-1 expression had a better prognosis than patients with high Trx-1 levels [79]. Cholangiocarcinoma (CCA) is a major health problem in north-eastern Thailand. Therefore, studies have been conducted to identify protein biomarkers for early detection of CCA. The levels of Trx and cadherin-related family member 2 (CDHR2) were significantly different in the serum from CCA patients compared with the control group [80]. Since diffuse malignant peritoneal mesothelioma (DMPM) is an aggressive malignant tumor with limited response to cytoreductive surgery combined with intraperitoneal chemotherapy, early diagnosis is very important. In general, serum levels of Trx were higher in DMPM patients compared with the control population, recommending serum Trx concentration as a useful diagnostic marker for DMPM [81]. Further promising results have been provided by studies analyzing the clinical and prognostic significance of Trx-1 in patients with gastric cancer (GC), associating poor prognosis with elevated Trx-1 expression levels [82]. 

### 3.3. Urogenital

Ribback and colleagues investigated the prognostic role of Trx-1 in clear cell renal cell carcinoma (ccRCC) and showed that cytoplasmic expression of Trx-1 was associated with poor prognosis. Otherwise, high Trx-1 levels were associated with less advanced clinicopathological features [83].

Studies in prostate cancer have shown that Trx dysfunction leading to redox changes also plays an important role in disease progression and response to therapy [84].

### 3.4. Hemato-Oncology

Trx-1 levels are elevated not only in solid tumors but also in hematologic malignancies (for overview see [85]).

Using immunohistochemical staining, Li and colleagues found that diffuse large B-cell lymphomas (DLBCL) have higher Trx-1 expression than normal B-cells [86], and that increased Trx expression is associated with poor progression-free survival [86,87]. Interestingly, Kari and colleagues observed that strong Trx-1 expression sensitized cells to etoposide, suggesting that Trx-1 could be used as a predictive biological marker for selecting patients who might benefit from the addition of etoposide to R-CHOP immunochemotherapy [88]. Shao et al. demonstrated that Trx-1 levels were variable in children with acute T-cell lymphoblastic leukemia and that these cells had higher Trx-1 levels associated with higher white blood cell counts [89]. 

## 4. Trx as a Predictive Biological and Prognostic Marker in Non-Cancer Diseases

### 4.1. Autoimmune and Inflammatory Diseases

Patients with rheumatoid arthritis (RA) had higher Trx plasma samples compared to healthy control patients, and disease activity correlated with Trx expression. In addition, plasma Trx levels correlated significantly with urinary excretion of 8-hydroxy-2′-deoxyguanosine (8-OHdG) [90]. Trx in the serum is also a prognostic value in patients with sepsis. It has been demonstrated that an early increase in serum levels of Trx-1 can predict 28-day mortality in sepsis patients in the intensive care unit (ICU) [91].

### 4.2. Lung

Bronchial asthma is a chronic inflammation of the airways. To clarify the clinical significance of Trx in the pathogenesis of asthma, blood samples were collected from patients with bronchial asthma with or without attack. Elevated Trx levels were found in the sera of patients with asthma attacks compared with those in the asymptomatic phase. Trx concentrations are related to the state of asthma exacerbation and allergic inflammation [92]. Trx concentrations in the bronchoalveolar lavage (BAL) fluid from patients who rejected a lung graft were significantly higher compared with those who did not, suggesting that Trx concentrations in BAL after lung transplantation may serve as a biomarker to assess the severity of graft rejection [93].

### 4.3. Abdominal Disease

Serum Trx-1 levels have been reported to correlate significantly with the severity of acute pancreatitis (AP). Trx-1 levels were significantly elevated in patients with severe AP compared with patients with mild AP. Therefore, Trx-1 could be used as a biomarker for assessing the severity of AP associated with oxidative stress [94].

In addition, Trx is a promising biomarker for the progression of hydronephrosis in children. One study reported that Trx is elevated in the serum and urine from children with hydronephrosis in comparison with healthy controls, and that the concentrations are related to the severity of the disease [95].

### 4.4. Cardiovascular

In addition, plasma Trx levels can be used to further explore and understand post-cardiac arrest syndrome. Trx levels increase early after cardiac arrest, and the most severe patients have the highest Trx levels [96]. 

Patients with cardiac events had significantly higher plasma Trx-1 concentrations than patients without cardiac events. A multivariate Cox proportional hazard analysis showed that elevated Trx-1 levels were independently associated with poor patient outcome [97].

Because some patients with coronary artery diseases (CAD) had elevated homocysteine and Trx levels, it was hypothesized that homocysteine, Trx levels, and CAD were associated. Analysis showed that the CAD groups had significantly elevated increased homocysteine levels but no Trx activity compared with the non-CAD group. Trx activity correlates closely with the extent and severity of CAD [98].

### 4.5. Cerebrovascular

To evaluate the potential diagnostic and prognostic role of Trx in acute ischemic stroke (AIS), patients’ serum Trx levels were measured. Several studies have shown that a high serum Trx level is associated with a better outcome in AIS patients [99], suggesting that Trx level may serve as a prognostic marker in patients with AIS [100,101].

In addition, serum Trx concentrations were elevated in patients with intracerebral hemorrhage (ICH) or subarachnoid hemorrhage (aSAH) compared with healthy controls. Trx concentrations were closely related to hemorrhagic severity and long-term mortality, suggesting that Trx could be used as a potential prognostic biomarker [102,103].

### 4.6. Trauma

A Swedish observational study of plasma samples from trauma patients showed an increase in Trx compared to healthy volunteers. In addition, Trx concentrations were associated with trauma severity and poor outcome in patients with severe traumatic brain injury (STBI). Already on the first day, plasma Trx concentrations were elevated in patients who later developed post-injury sepsis, suggesting that Trx may act as a biomarker in trauma patients [104,105]. 

### 4.7. Aging

Trx appears to be a putative biomarker in age-related immune decline, as shown by the MARK-AGE study. The study confirmed that there is an inverse correlation between surface Trx-1 expression and age. However, analysis in a larger patient cohort of patients is needed to confirm this finding [71].

Chronic mountain sickness (CMS) is a progressive incapacitating syndrome induced by lifelong exposure to hypoxia. To identify potential biomarkers in the plasma between the CMS and non-CMS groups, a search was conducted for differentially expressed genes, and Trx-1 was discovered, among others. This study provides insights into the pathogenesis of CMS to develop new treatments [106].

## 5. Trx-1 in Health and Disease—Potential Therapeutic Approaches

**Figure 5:**
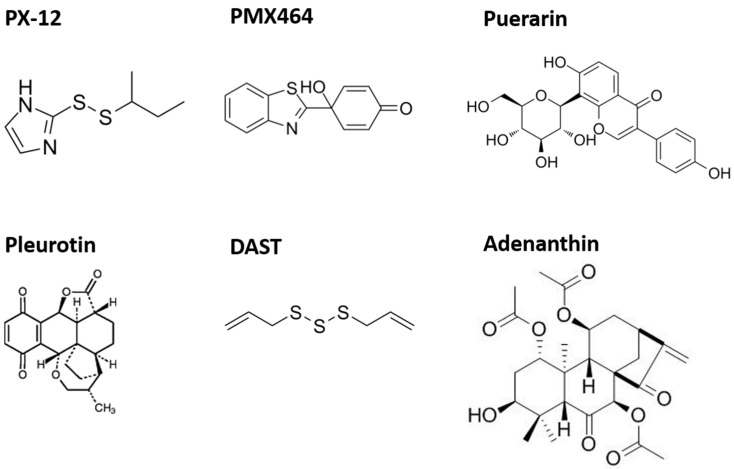
Overview of selected inhibitors of the Trx(-system).

Trx-1 is essential for proper organ function, but alterations in Trx-1 expression or activity, as well as protein–protein interactions, can cause a switch from a physiologic state of cells and organs to various pathologies. Therefore, therapeutic approaches are increasingly being developed to find new selective inhibitors targeting this protein (Figure 5). In general, the majority of available inhibitors of the Trx system target TrxR. However, specific Trx inhibitors have been tested in humans. 

### 5.1. PX-12 (1-Methylpropyl 2-Imidazolyl Disulfide)

PX-12 was the first Trx-1 inhibitor to enter clinical trials as an anticancer agent [82,107,108,109,110,111,112,113,114,115,116].

PX-12 is tolerated up to a dose of 226 mg/m^2^ by a 3 h infusion in patients with solid tumors [112], while patients with advanced refractory cancers tolerated higher infusion [112,117]. In general, Trx-1 inhibition seems to have a promising therapeutic potential. Using a tumor xenograft model and the Trx-1 inhibitor PX-12, the relationship to Trx-1 and vascular endothelial growth factor (VEGF) levels should be addressed. The data demonstrated a rapid decrease in the average tumor blood vessel permeability, as well as a decrease in tumor and tumor-derived VEGF in plasma after treatment with PX-12. However, Trx-1 showed a rapid decline within 2 h following PX-12 administration, which was maintained for 24 h. The decrease in tumor vascular permeability caused by PX-12 administration coincided with a decrease in Trx-1 and VEGF [118]. 

A decrease in vascular permeability in tumor xenografts [118] gives rise to the question of whether PX-12 could be also effective in the early stage of focal cerebral ischemia by decreasing blood–brain barrier (BBB) disruption. Beyond this, the question arises whether PX-12 inhibition could be affected by vascular endothelial growth factor (VEGF), which itself interacts with the Trx-1 system. The data showed that PX-12 is effective in decreasing BBB disruption in the early stage of focal cerebral ischemia, but VEGF does not influence the action of PX-12 on BBB permeability [119].

Colorectal cancer (CRC) is mostly challenged by treatment failure and recurrence due to resistance to radiotherapy. In general, CRC tissues showed increased ALDH1L2 expression. Moreover, patients with high ALDH1L2 expression displayed poorer recurrence-free survival and overall survival compared to patients showing low ALDH1L2 expression [120]. It has also been demonstrated that ALDH1L2 interacts with Trx and, thereby, regulates the downstream NF-κB signaling pathway. Beyond this, application of PX-12 overcame radioresistance due to decreased ALDH1L2 expression. Finally, the data suggested that simultaneous application of Trx-1 inhibitors and radiotherapy might ameliorate clinical outcomes of patients with CRC with low ALDH1L2 levels [121].

### 5.2. Quinols

In general, heteroaromatic quinols show potent antitumor activity against various cancer cell lines, as well as inhibited tumor growth in different xenograft models. Mass spectrometry analysis confirmed the binding between reduced Trx-1 and quinol analogues resulting in reduced enzyme activity [122], thereby leading to speculation that quinol analogues react with Trx in a double Michael addition.

### 5.3. PMX464 (4-(Benzothiazol-2-yl)- 4-Hydroxycyclohexa-2,5-dienone—Previously AW464; [123])

In addition to PX-12, another known Trx inhibitor is PMX464 [124]. Studies on colorectal and breast cancer cell lines have demonstrated that PMX464 enhanced hypoxic anti-proliferative effects in the cell lines and endothelial cells, as well suggesting that PMX464 may be a promising chemotherapeutic drug with anti-angiogenic activity [125]. Further analysis demonstrated that PMX464 inhibits Trx-1 function without affecting its expression. Its inhibition correlated with decreased proliferation and survival. Therefore, the Trx-1 system is an important target in tumor cells and can be inhibited by PMX464 [126].

Additionally, oxidation of thiols in cell surface proteins, including the collagen receptor GPVI and the von Willebrand factor receptor, is affected by PX464. These results revealed a novel role for Trx in regulating platelet function and thrombus formation, suggesting a potential opportunity for repurposing this Trx inhibitor as an antiplatelet agent [127]. 

### 5.4. Secondary Metabolites

It is commonly known that secondary metabolites from plants and fungal affect the Trx system [128,129,130]. 

#### 5.4.1. Puerarin

Gegen, the root of *Pueraria lobata (Willd.) Ohwi*, is used as a traditional Chinese medicine with various medicinal purposes. The major bioactive ingredient isolated from Gegen is called puerarin [131], and it is widely used for treatment of cardiovascular and cerebrovascular diseases, neurodegenerative diseases, and cancer [132].

Administration of puerarin within 4 h of spinal ischemia-reperfusion rescued ischemic reperfusion damage injury by induction of Trx transcription and reduction in apoptosis [133]. 

Furthermore, Dil-oxLDL uptake is inhibited by puerarin in RAW264.7 cells and in primary macrophages. Moreover, in apoE-/- mice, combination treatment with PX-12 or Trx-1 siRNA demonstrated that reduced lipid uptake by puerarin requires Trx-1 inhibition in vivo. This finding suggests that puerarin might be an effective therapeutic in the prevention of atherosclerosis by affecting Trx-1 [134].

#### 5.4.2. Pleurotin

Pleurotin is a promising basidiomycete metabolite that was first isolated from *Pleurotus griseus* Peck [135]. Meanwhile, pleurotin is classified as *Hohenbuehelia grisea* (Peck) Singer. The metabolite showed various functions, including anti-cancer effects [136], which can be attributed to its inhibition of the Trx-system [137]. In vitro studies on human breast or colon cancer cell lines revealed that treatment of cells with pleurotin inhibited hypoxia-induced upregulation of HIF-1α. Furthermore, treatment with pleurotin under hypoxic conditions decreased HIF-1-trans-activating activity, VEGF formation, and inducible nitric oxide synthase. It is suggested that Trx-1 inhibitors contribute to the growth-inhibitory and antitumor effects of these agents by inhibiting HIF-1α [138].

Furthermore, the question arises whether thioredoxin inhibitors can act as antifungals. To test this hypothesis, the activity of pleurotin against the dermatophyte *T. mentagrophytes* and *Candida albicans* was tested. In vitro and ex vivo analyses showed that pleurotin inhibits the growth of the dermatophyte but has no effect on *Candida*. Therefore, Trx inhibitors should be further developed and optimized for their use as antifungals [139].

#### 5.4.3. Adenanthin 

It is known that adenanthin can inhibit the enzymatic activities of peroxiredoxins (Prdx), which have a thioredoxin fold. Therefore, it stands to reason that adenanthin also inhibits the Trx- system. Indeed, in vitro analyses have shown that adenanthin decreases Trx activity by binding to Trx-1. Thus, adenanthin may be a new potential therapeutic agent for diseases in which aberrant activity of Prdx or the Trx-system is involved in pathogenesis [140].

#### 5.4.4. Diallyl Trisulfide (DAST)

Diallyl trisulfide (DATS) is one of the major sulfur-containing compounds in garlic oil. Through Michael addition, DAST conjugated directly to the Cys32 and Cys35 residues of Trx-1, resulting in inhibition of Trx-1 activity. 

In patients with triple negative breast cancer (TNBC), metastasis is the main cause of high mortality. It has been suggested that Trx-1 plays an essential role in breast cancer metastasis. It was found that the production of the reduced form of Trx-1 was dramatically reduced by DAST in vitro. In vivo analysis showed that DAST administration suppressed spontaneous and experimental metastasis in nude mice. Reduced Trx expression was also observed in primary tumors after DAST administration. In conclusion, targeting the Trx system with DATS is a promising strategy for the treatment of TNBC metastasis [141].

Glioblastoma (GBM) patients usually receive radiotherapy as the standard treatment. However, resistance to radiotherapy remains a major challenge. Previously, it was shown that DATS could directly conjugate to the redox-active cysteine residues in Trx-1, thereby inhibiting its activity. Consequently, ROS production is induced. Furthermore, DAST has been shown to enhance irradiation (IR)-induced ROS accumulation, apoptosis, and DNA damage, and inhibit tumor growth of GBM cells as well. These observations suggest the utility of DATS in sensitizing radiotherapy to GBM patients [142]. 

#### 5.4.5. Quinones (P-Benzoquinone, BQ)

Quinones are ubiquitous biological pigments found in various biological organisms. In most cases, they occur in nature in forms such as benzoquinones, naphthoquinones, anthraquinones, and polycyclic quinones. In general, quinones can alter molecules through redox reactions that produce radicals and through covalent adduction. Mass spectrometric analysis showed that BQ forms adducts with all Cys residues of Trx-1, resulting in a loss of enzyme activity. It was also shown that the reaction of BQ with Trx leads to activation of the ASK1/p38 MAPK pathway and induction of apoptosis [143].

### 5.5. Administration of Trx-1

A number of preclinical studies have demonstrated that extracellular Trx has a cytoprotective function under oxidative and inflammatory conditions, with no evidence of adverse effects or adverse symptoms. In general, health benefits of plant food-based diet could be associated with integrated antioxidant and anti-inflammatory mechanisms [144,145]. For example, fermented foods and beverages have shown a strong impact on human gut microbiota. For example, papaya, the fruit of the *Carica papaya* plant, is commonly used as a medicinal fruit [145]. However, there is also evidence for the use of the fermented form of this fruit. 

For example, in a clinical study of cirrhotic patients given fermented papaya, lower Trx levels, improved redox balance, and reduced TNF-α production by monocytes were observed [146].

Interestingly, rats orally administered Trx from *Saccharomyces* showed beneficial effects on gastric mucosa by upregulating genes related to tissue repair, suggesting that administered yeast Trx may protect the gastric mucosa [147].

In addition, sodium butyrate (NaB), a short-chain fatty acid produced by bacterial fermentation of indigestible dietary fiber, has been shown to have an anti-tumor effect in CRC. Treatment with NaB inhibited cell growth and decreased Trx-1 protein expression in CRC cells and also induced apoptosis in CRC cells as well [148].

### 5.6. Recombinant Trx-1

Excessive neutrophil elastase activity in the airways of cystic fibrosis patients (CF) leads to progressive lung damage. It has been speculated that altering the enzymatic activity of elastase may have potential therapeutic properties. In a comparative study, *E. coli* Trx and human recombinant Trx (rhTrx) were tested for their effects on elastase activity. Reduced rhTrx inhibited purified elastase and soluble CF sputum elastase. These findings make these compounds potential therapies for CF [149]. Furthermore, in a lung injury rat model, continuous infusion of rhTrx was shown to significantly reduce neutrophil infiltration in the airways [150]. In addition, continuous administration of rhTrx significantly suppressed the percentage of neutrophils in bronchoalveolar lavage fluid. Histological examination also showed that rhTrx decreased neutrophil infiltration in the lung tissue [151]. In addition, studies in mice investigating the effect of Trx on ultraviolet (UV)-B-mediated inflammatory and apoptotic responses showed that administration of rhTrx attenuated acute skin inflammatory conditions such as skin erythema and swelling [152].

In addition, studies in mice showed that recombinant Trx may also be effective in preventing N-acetyl-p-aminophenol (APAP) induced liver injury. It was observed that injection of rhTrx significantly reduced liver injury and mortality. Moreover, pretreatment of mice with rhTrx-1 also showed such beneficial effects. Recombinant human Trx decreased the expression of RIP-3, resulting in less necroptosis [153].

The antioxidant Trx-1 has been shown to protect cells from OS-induced cell death. In one study, the effect of Trx on oxidized low-density lipoprotein (ox-LDL-)-induced macrophage foam cell formation and apoptosis was investigated by using human recombinant Trx-1 on stimulated macrophages. The data showed that human recombinant Trx-1 significantly inhibited ox-LDL-induced ROS production and apoptosis, suggesting that human recombinant Trx-1 may function as a novel therapeutic target in the prevention or treatment of atherosclerosis [154]. 

### 5.7. Human Serum Albumin-Trx Fusion Protein (HSA-Trx)

In general, Trx may offer great potential as a therapeutic agent. However, due to its small molecular weight, Trx is largely excreted via glomerular filtration. The half-life of Trx is extremely short (about 1 h). Therefore, the poor blood retention property of Trx should be improved. Albumin fusion of Trx has been reported to have great clinical applications.

Ikuta and colleagues generated a fusion protein between HSA and Trx and demonstrated similar plasma concentrations and organ distribution of the fusion protein as human albumin. Surprisingly, the fusion protein showed better results than Trx in some assays [155]. Moreover, in a mouse model, HSA-Trx was shown to suppress ROS production and neutrophilic inflammation, thereby preventing acute lung injury caused by urban aerosols. Thus, HSA-Trx could be a potential drug candidate for the prevention of lung injury [156]. Subsequently, it was observed that HSA-Trx could inhibit ROS production and generation of 8-OHdG in a lung model [157]. 

Furthermore, HSA-Trx showed positive effects in inhibiting oxidative stress in the kidney, thereby preventing kidney damage in a rat model of ioversol-induced contrast-induced nephropathy (CIN). Thus, long-acting HSA-Trx is a new potential therapeutic agent for the effective prevention of CIN [158]. Furthermore, studies on the effect of the fusion protein on Cu^2+^/Zn^2+^-induced neurotoxicity showed that metal iron-induced ROS production and the expression of oxidative stress-related genes were suppressed by HSA-Trx. These results suggest that HSA-Trx may be a promising therapeutic agent for the treatment of refractory neurological diseases [159].

Preventing the transition from acute kidney injury (AKI) to chronic kidney disease (CKD) remains highly desirable. Analysis of mice with AKI and CKD produced by renal ischaemia–reperfusion (IR) showed that HSA-Trx ameliorated tubular injury and fibrosis by suppressing renal oxidative stress, pro-inflammatory cytokine production and macrophage infiltration. In addition, HSA-Trx also inhibited apoptosis in renal tubular cells. Thus, HSA-Trx modulated oxidative stress and inflammation and should be considered for the treatment of the transition from AKI to CKD [160].

### 5.8. In Vitro or In Vivo Manipulation of Trx in Disease

Since the cellular redox state is known to regulate the physiological state of cells and organs, several in vitro and animal studies have been conducted to investigate the effects of overexpressing Trx-1 or reducing Trx-1 gene expression.

#### 5.8.1. In Vitro or In Vivo Deficiency of Trx

Since Trx-1 or Trx-2 deficiency is embryonically lethal in mice [13,14], few studies have been performed in mice with an organ specific knock-out of Trx-1 (KO-Trx-1) or dominant negative Trx-1 transgenic mice were carried out. Das and colleagues conducted one of the first studies using Trx-1-deficient mice. They generated mice lacking Trx-1 in the lungs using a dominant–negative construct. At normal oxygen levels, these mice showed a decrease in mitochondrial energy metabolism and an increase in proinflammatory cytokine production. However, Trx-1-deficient mice showed significant mortality under hyperoxia conditions for several days. These results suggest that Trx plays an important role in maintaining redox homeostasis in the lung, as well as protecting against pneumonia [161]. Oka and colleagues investigated the pathophysiological role of endogenous Trx-1 using heart-specific Trx-1 knockout mice. Surprisingly, the transgenic mice were viable but developed various disease symptoms including heart failure, hypertrophy, and increased fibrosis. Using RNA sequencing, they found that these mice had down-regulation of genes involved in energy production. In addition, these mice exhibited abnormal mitochondrial morphology caused by mTOR regulation [162].

Further promising results were obtained by Jabber and colleagues, who analyzed the effects of Trx-1 deficiency on the regulation of hematopoietic stem/progenitor cells (HSPCs) under stress, such as radiation injury. They found that Trx-1 deficiency makes HSPCs more sensitive to radiation and impairs the reconstitution and differentiation of HSPCs. Using CRISPR/Cas9 knock-out, they generated an EML mouse hematopoietic stem/progenitor cell line lacking Trx-1. These cells were used to analyze the effect of Trx-1 on apoptosis. The enhanced apoptosis pathway and the resulting impaired reconstitution of HSPCs lead to the conclusion that Trx-1 is necessary for protection against radiation-induced injury and hematopoietic recovery, which is of great interest for clinical applications [68].

#### 5.8.2. In Vitro or In Vivo Overexpression of Trx-1 

Furthermore, pancreatic beta cells overexpressing Trx-1 showed positive effects on the progression of type 2 diabetes mellitus, and Trx-1 expression had a positive effect on body weight gain and insulin levels. These results demonstrate that thioredoxin-1 can improve beta cell survival and function [163]. Furthermore, overexpression of Trx-1 significantly improved the development of diabetic nephropathy in streptozotocin-induced diabetic mice. Diabetic mice overexpressing Trx-1 showed lower urinary albumin excretion and fewer pathological changes, including less tubular damage, compared to diabetic control animals. In summary, these results suggest that OS plays an important role in the development of diabetic nephropathy and that progression may be enhanced by modulation of Trx-1 expression [164].In studies using the same mice to investigate the effects of diabetes mellitus on the development of osteopenia and bone fractures, diabetic mice overexpressing Trx-1 were found to have similar body weight and kidney function to wild-type diabetic control mice. However, oxidative DNA damage, as measured by urinary 8-OHdG excretion and 8-OHdG staining in bone tissue, is decreased in Trx-1-overexpressing animals, suggesting that increasing Trx expression may be a potential therapeutic approach for diabetes-induced osteopenia [165]. In addition, studies analyzing neuronal degeneration or photoreceptor degeneration have shown better results. After ischaemia–reperfusion, mice overexpressing Trx-1 had a lower number of fluorojade B-positive neurons and a lower number of caspase-3- and TUNEL-positive cells compared to control mice [166]. Similar results were obtained in tubby mice overexpressing human Trx-1. Normally, tubby mice are characterized by sensorineural deafness and retinal dystrophy. However, overexpression of human Trx-1 reduced photoreceptor loss by enhancing neurotrophic factors such as BDNF and GDNF and activating Akt signaling, while inhibiting apoptosis signaling pathways [167].

Trx-1 is thought to play an important role in the pathogenesis of cardiovascular disease. To clarify whether Trx-1 is also involved in sepsis-induced myocardial dysfunction, heart-specific Trx-1 overexpressing transgenic mice were generated and subjected to surgical procedures. In addition to prolonging the average lifespan of the mice, Trx-1 overexpression also showed preserved contractile reserve and enhanced induction of mitochondrial biogenesis as well as increased antioxidant capacity [168].

### 5.9. Therapeutic Agents

The rheumatic drug KE-298 (2-acetylthiomethyl-4-(4-methylphenyl)-4-oxobutanoic acid) influenced the secretion of Trx and increased the level of intracellular glutathione in human monocytes and T cells, thereby acting efficiently in the treatment of RA [169].

In various cancers such as T-cell lymphomas and leukemia, constitutively active NFκB promotes survival. Dimethyl-fumerate (DMF) counteracts NFκB by suppressing Trx-1, leading to increased ripoptosome-induced cell death. These findings offer a new approach for the treatment of NFκB-dependent tumors [170]. Studies using a pheochromacytoma cell line (PC12 cells) showed that 1-metyhl-4-phenylpyrimidinum ion (MPP(+)) induced cell death by suppressing of Trx expression. Moreover, overexpression of Trx attenuated MPP(+)-induced neurotoxicity in these cells. These observations provide new insights for potential new therapeutic agents in Parkinson’s disease [171].

Studies in liver cells have shown that sorafenib has scavenging properties and downregulates Trx-1 expression. Experimental models of HCC showed that the antitumor properties of sorafenib are related to a decrease in Trx-1 expression, S-nitrosation (SNO)-CD95 and caspase-8 activity. Furthermore, it is speculated that a combination of sorafenib with Trx-1 inhibitors should be considered for therapies against advanced HCC [172,173].

## 6. Conclusions

Trx-1 is one of the most essential proteins for proper organ function. However, there are several mechanisms inducing a switch from the physiologic state of cells and organs to diverse pathologies. For example, Trx gene expression is modulated by TFs such as Nrf2 that bind at specific binding sites in the promotor region [31]. In addition, Trx activity can be modulated by post-translational modifications such as phosphorylation of Trx at T100, which promotes apoptosis in cancer cells [45]. In addition, S-nitroylation of Trx at Cys62, Cys69, or Cys73 is known to be required for full redox activity. Additionally, glutathionylation of Trx at Cys73 prevents dimer formation, resulting in abolition of enzymatic activity [46]. Another possibility for modulating Trx activity are inhibitors such as Txnip, PX-12, or even secondary metabolites. Modulation of Trx-1 expression or Trx activity can therefore make the difference between health and disease.

In summary, a deeper understanding of Trx-1 has the potential to open up new therapeutic applications. By exploring the importance and potential of this protein in this review, researchers may be able to develop new diagnostic applications or treatments for a variety of diseases. Therefore, further research into Trx-1 is crucial to improve our understanding of its role in human health and disease.

## Data Availability

Not applicable.
